# Telomere Recombination Preferentially Occurs at Short Telomeres in Telomerase-Null Type II Survivors

**DOI:** 10.1371/journal.pone.0090644

**Published:** 2014-03-03

**Authors:** Xiao-Hong Fu, Yi-Min Duan, Yu-Ting Liu, Chen Cai, Fei-Long Meng, Jin-Qiu Zhou

**Affiliations:** 1 The State Key Laboratory of Molecular Biology, Shanghai Institute of Biochemistry and Cell Biology, Shanghai Institutes for Biological Sciences, Chinese Academy of Sciences, University of Chinese Academy of Sciences, Shanghai, China; 2 School of Life Science and Technology, Shanghai Tech University, Shanghai, China; Universita’ di Milano, Italy

## Abstract

In telomerase negative yeast cells, Rad52-dependent recombination is activated to maintain telomeres. This recombination-mediated telomere elongation usually involves two independent pathways, type I and type II, and leads to generation of type I and type II survivors. It remains elusive whether the recombination-mediated telomere elongation prefers to take place on shorter or longer telomeres. In this study, we exploited the *de novo* telomere addition system to examine the telomere recombination event in telomerase negative cells. We show that recombination preferentially occurs on shorter rather than longer telomeres in both pre-survivors and established type II survivors. In type II survivors, the short VII–L telomeres could invade either terminal TG_1–3_ sequence or short tracts of TG_1–3_ sequence in subtelomeric Y′-X and Y′-Y′ junction to initiate recombination. Unexpectedly, short VII–L telomere recombination still takes place in type II survivors lacking either Rad50 or Rad59, which are required for type II survivor generation in senescing telomerase-null cells. Our results support the notion that Rad50 and Rad59 are not essential for the maintenance of type II survivors once established.

## Introduction

Telomeres are the physical ends of eukaryotic chromosomes, and consist of telomeric DNA of repetitive sequence and protein complexes. They prevent chromosomal ends from degradation or fusion, and are essential for chromosomal integrity and genome stability in eukaryotes [1]. In budding yeast *Saccharomyces cerevisiae*, telomeric DNA consists of ∼300±75 bps of TG_1–3_/C_1–3_A repeats with a protruding G-rich single-stranded 3′ overhang (G-overhang) [2], [3]. Immediately internal to the telomeric TG_1–3_ sequence, there are telomere associated sequences/elements, called X and Y′ elements [4]. Y′ elements are either 6.7 or 5.2 kb long [5]. Some telomeres have no Y′ element, and some have as many as four copies of Y′ elements. Short tracts of TG_1–3_ sequence are often found between two Y′ or X and Y′ elements. Like most of the eukaryotes, budding yeast elongates its telomeric DNA through a specialized reverse-transcriptase, telomerase, which adds telomeric DNA sequences to the 3′ G-overhang using its intrinsic RNA template [6], [7]. The telomerase holoenzyme in *S. cerevisiae* is composed of the catalytic subunit Est2, the RNA template subunit Tlc1 and two accessary subunits, namely Est1 and Est3 [8]–[12]. Deletion of any of the telomerase subunits causes gradual telomere shortening and cellular senescence [10], [13]. Most of the telomerase-null senescing cells eventually die, while a very small portion of cells can overcome the crisis and remain viable. These cells are called “survivors”, whose chromosome ends are lengthened by homologous recombination [14], [15]. According to different telomeric DNA structures, survivor cells are roughly divided into two categories, type I and type II survivors [16], [17]. The recombination-mediated telomere elongation (namely “alternative lengthening of telomeres” or ALT) is also found in mammalian cells. For example, approximately 15% of immortalized human tumor cells apply the ALT mechanism to maintain telomeres [18], [19].

The generation of telomerase-null survivors appears to involve multiple steps of repairing shortened telomeres through homologous recombination. The type I survivors amplify subtelomeric Y′ elements and short terminal telomeric TG tracts. The process of amplifying Y′ elements is dependent on the canonical homologous recombination proteins Rad51, Rad52, Rad54, Rad55 and Rad57 [20]. The type II survivors generate long heterogeneous telomeric TG_1–3_ tracts, and this process requires a subset of DNA repair proteins, such as Rad52, Rad59, Sgs1 and the Mre11-Rad50-Xrs2 (MRX) complex, as well as Mec1 and Tel1 kinases [20]–[23]. The type II survivors of yeast are thought to resemble the ALT cells in mammals because both have long and heterogeneous telomeric repeats [15]. The type I survivors have extremely short TG tracts which affect the cell cycle progression and make the cells grow poorly [14], [24]. In contrast, the type II survivors grow at a rate similar to telomerase proficient cells. Therefore, type II survivors eventually overtake type I survivors in liquid-grown cultures [15], [20].

Previous studies have shown that in wild-type yeast cells, telomerase usually acts on and preferentially elongates short telomeres [25]–[28]. In the absence of telomerase, Rad52-dependent homologous recombination is believed to be the major pathway that repairs telomeres. However, it remains elusive whether the recombination machinery also prefers shorter to longer telomeres. Recently Chang et al reported that long telomeres are the more preferable targets of recombination during survivor emergence [29], while other two independent studies suggested that only short telomeres engage in recombination in either pre-survivors or survivors [21], [30]. In this study, we examined the recombination-mediated telomere replication events in telomerase-null cells, and observed a preference for the extension of short telomeres in both pre-survivors and established type II survivors. In type II survivors, the short VII–L telomeres could invade either terminal TG_1–3_ sequence or short tracts of TG_1–3_ sequence in subtelomeric Y′-X and Y′-Y′ junction to initiate recombination. Surprisingly, unlike Rad52, Mre11/Rad50/Xrx2 complex and Rad59, which are required for type II survivor generation, greatly affect, but are not absolutely required for the *de novo* telomere elongation through recombination in type II survivor cells.

## Materials and Methods

### Yeast Strains and Plasmids

Strains used in this work are derived from UCC5706 (*MATa-inc ura3-52 lys2-801 ade2-101 trp1-Δ63 his3-Δ200 leu2-Δ1:LEU2-GALHO VII*–*L::ADE2-TG-HO site-LYS2 rad52::hisG*) (kindly provided by Dr. Daniel Gottschling) [31]. A fragment (TRP1-TG-HO cassette) containing a *TRP1* selection marker, 81 bp or ∼300 bp TG sequence and a 30 bp HO recognition site was generated from the modified pSD158 plasmid (kindly provided by Dr. Daniel Gottschling). After reintroduction of a *RAD52* CEN plasmid, pRS316-*RAD52*, the TRP1-TG-HO cassette was integrated into the *ADH4* locus to make the TG81 and TG300 strains.

### Telomere Sequencing

Telomere PCR and following sequencing were performed as described in [32].

### Telomere Southern Blot

Genomic DNA was digested with XhoI (for massive telomere detection), SpeI/EcoRV (for single telomere detection of chromosome VII–L) or SpeI/EcoRV/NspI/ScaI (for detection of Y′-containing recombination products of chromosome VII–L), separated on 1% agarose gels, transferred to Hybond-N+ membrane (GE Healthcare), and hybridized to a TG_1–3_, Y′ or TRP1 probe.

### HO Induction Assay

Cells were grown in yeast complete media lacking both uracil and lysine (YC/Ura^−^Lys^−^) plus 2% raffinose (Sigma), diluted into YC/Ura^−^ plus 2% raffinose and cultured to logarithmic phase. Galactose (Sigma) was added to a final concentration of 3%, and cells were cultured for an additional 24 hrs. Samples were processed for plating or dotting assays. Both liquid and solid cultures were grown at 30°C.

### Dotting, Plating and Viability Assays

Cell cultures were diluted with sterile water to a concentration of OD_600_ ∼0.3, and 3 µl of each 5-fold serial dilutions was dotted on YC plates. Plates were incubated at 30°C for 2 to 3 days and then photographed. 100 µl of the 5^th^ dilution sample (in dotting assay) was plated. YC/Ura^−^Lys^−^ (uninduced total) was used for uninduced samples, and YC/Ura^−^ (induced total) and YC/Ura^−^Lys^−^ (induced uncut) was used for induced samples. Plates were incubated at 30°C for 2 to 3 days and colony numbers were counted. The viability was calculated using the formula: Viability = (Induced total - Induced uncut)/Uninduced total.

## Results

### Shorter Telomeres are Subjected to Elongation in the Senescing Telomerase-null Cells

**Figure 1:**
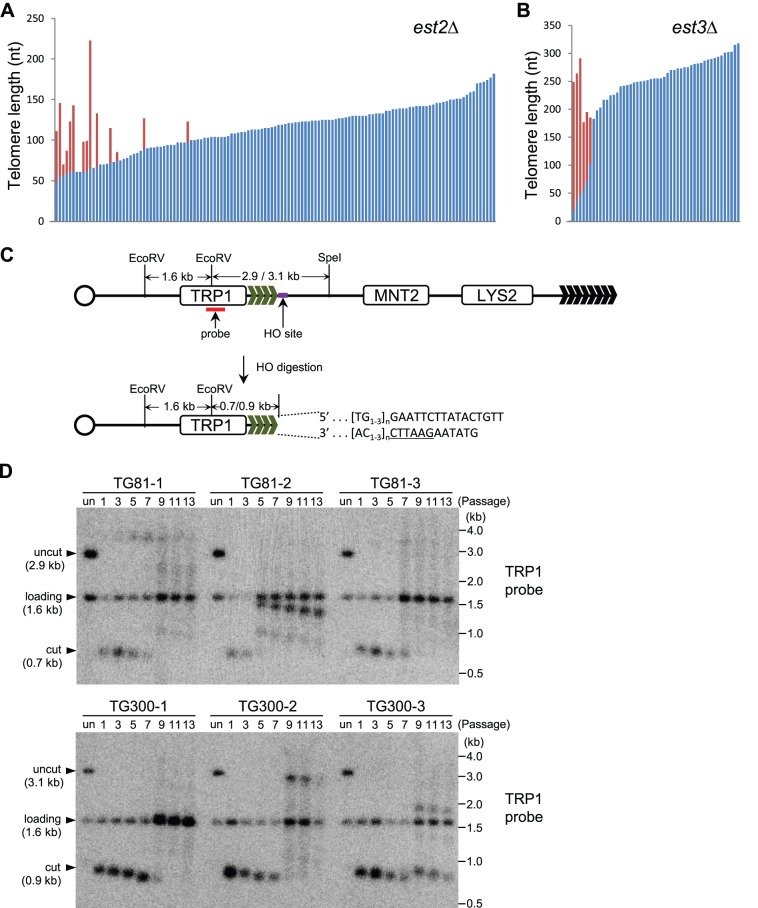
Recombination occurs on shorter telomeres in the senescing telomerase negative cells. Telomere I–L sequencing analysis of (A) *est2*Δ and (B) *est3*Δ cells. Each bar represents one sequenced telomere. Constant sequence is marked in blue and divergent sequence is marked in red. (C) Schematic representation of HO induction assay. The *LYS2* and *MNT2* genes serve as genetic markers to monitor the HO cleavage. The HO endonuclease recognition site and the TRP1 probe used to detect the TG_1–3_/HO end are shown in purple and red, respectively. The EcoRV and SpeI sites indicated are used for Southern digestion, which generates a fragment of ∼2.9 kb (TG81 strain) or ∼3.1 kb (TG300 strain). The EcoRV site within the *TRP1* gene is located 600 bp away from the inserted TG seed. The fragment between the two EcoRV sites (∼1.6 kb) serves as a loading control. This figure is not precisely drawn to scale. (D) Southern blot examination of the HO induction assay in senescing *tlc1*Δ cells. HO induction was performed immediately after *TLC1* deletion. Three single clones of each strain (TG81 or TG300) were inoculated and passaged for 13 times (labeled on the top of each panel) in liquid. EcoRV and SpeI were used for Southern digestion and the TRP1 probe was used for hybridization. (“un”, uninduced control).

In budding yeast *Saccharomyces cerevisiae*, inactivation of telomerase causes telomere shortening and cellular senescence [9], [10], [13]. In order to know the change of telomere sequence in the senescing telomerase-null cells, we obtained an *est2*Δ spore by dissecting a tetrad derived from the heterozygous *est2*Δ/*EST2* strain, and passaged the spore for about 60 generations, a time point that survivor had not emerged yet. Then we analyzed the telomere sequences of chromosome I–L. 131 independent clones were subjected to telomere sequencing (Fig. 1A, Seq. S1). The sequencing results showed that telomere I–L of 14 clones contains divergent sequence (marked in red, Fig. 1A) [33]. These sequence divergences presumably resulted from recombination events because telomerase had been inactivated in the *est2*Δ cells. Interestingly, the recombination events appeared to take place only on some of the telomeres that were shorter than 100 bp, and similar results were obtained in *est3*Δ cells (Fig. 1B, Seq. S2). In the wild-type cells, telomere elongation presumably mediated by telomerase occurs on most of the telomeres [32]. This observation indicated that telomere recombination is in action in the senescing telomerase-null cells, but not efficient enough to repair all the short telomeres to prevent the cells from entering crisis and senescence. The extremely low emerging frequency of survivors could also be attributed to the insufficient recombination activity. We also noticed that the extent of new sequence added onto short ends seemed not to be greater than the lengths of non-recombined telomeres present in the cells. This may result from simply copying from another eroded short telomere rather than copying from a t-circle by a rolling circle mechanism, which could generate very long telomeres [34]. Furthermore, it seems that in *est3*Δ cells, recombination takes place on much shorter telomeres than that in *est2*Δ cells (Figs. 1A and 1B), possibly suggesting a protection role of Est2/Tlc1 in senescing cells.

To determine whether recombination-mediated telomere elongation prefers shorter telomeres, we exploited the *de novo* telomere addition system that was reported previously [31]. In our experiments, we chose 81-bp and 300-bp TG-tract to represent “short” and “long” telomeres. In the strain of TG81 or TG300, a *TRP1* selection marker, 81 bp or ∼300 bp (wild-type) telomeric “seed” and an HO endonuclease recognition site were integrated into the left arm of chromosome VII at the *ADH4* locus (Fig. 1C). When galactose is added into the culture, the HO gene under the control of a galactose-inducible promoter will be expressed. The HO recognition sequence is recognized and cut by the HO endonuclease, resulting in a 16 bp non-telomeric chromosome end including a TGTT 3′ overhang, which is adjacent to TG “seed” of either 81 or 300 bp (Fig. 1C). The fragment with native chromosome end is released, and the integrated telomeric “seed” is exposed (Fig. 1C). The exposed “seed” could be subjected to recombination upon resection of the remaining HO recognition sequence if telomerase is absent. Following these thoughts, we first deleted *TLC1* in the TG81 or TG300 strain using the plasmid integration approach [35]. Right after *tlc1*Δ colonies were formed on a selective plate, the *tlc1*Δ cells of three independent colonies were transferred into galactose-containing liquid medium to induce HO cutting and passaged to allow senescence to take place and type II survivors to arise (Fig. S1). The recombination events of telomere VII–L in the senescing *tlc1*Δ cells of TG81 and TG300 were examined by Southern blot analysis using a TRP1 probe. The genomic DNA was digested with EcoRV and SpeI. The HO-uncut fragments recognized by the TRP1 probe should be ∼2.9 kb and ∼3.1 kb in length in the TG81 and TG300 strains, respectively (Fig. 1D). After HO induction, the chromosome fragments containing *LYS2* would be lost, and the fragments recognized by the TRP1 probe should be shifted to ∼0.7 kb and ∼0.9 kb in the TG81 and TG300 strains, if there are no resection and recombination, which respectively shortens and lengthens the TG-tracts exposed (Fig. 1D). All the clones in both TG81 and TG300 strains did not grow on lysine minus plates (data not shown), indicating that they were auxotrophic for lysine, and that the HO digestion worked well. In the three clones of TG81 strain, the smearing hybridization signals of longer products indicated that the recombination occurred on telomere VII–L at the 5^th^, 5^th^ and 7^th^ passage respectively, whereas in the TG300 clones, the recombination took place on telomere VII–L at the 9^th^ passage (Fig. 1D). It seems that once the telomeres reach a critical length, recombination happens, so it takes more cell divisions for the telomeres to be trimmed to trigger recombination in the TG300 strain. During the process of survivor formation in the TG81 strain (TG81-2 and TG81-3), the bulk telomere recombination, compared with the recombination on telomere VII–L, occurred simultaneously with the lengthening of the short HO-cut telomeres, which is earlier than that in the TG300 strain (Figs. 1D and S1). These results suggest that the short VII–L telomeres in TG81 cells are not only extended sooner, but also induce a more rapid generation of type II survivors.

### Recombination Occurs on Shorter Telomeres in Type II Survivors

**Figure 2:**
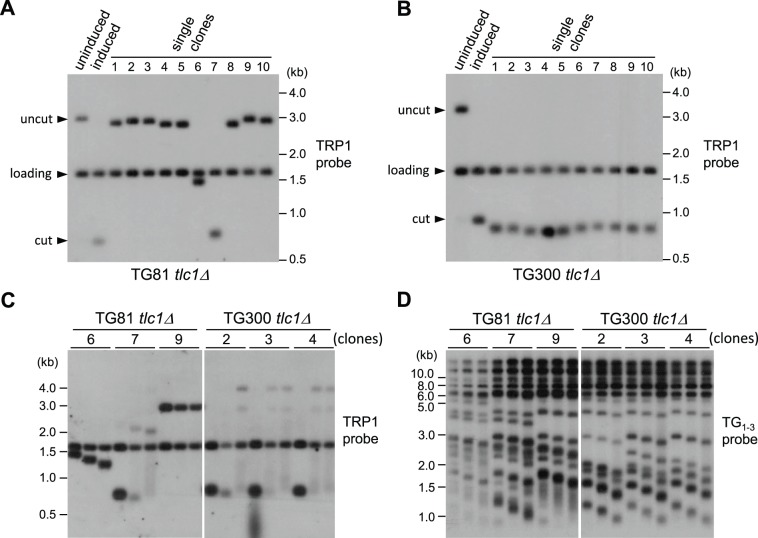
Shorter telomeres are preferentially elongated by recombination in type II survivors. Detection of VII–L telomere recombination in (A) TG81 and (B) TG300 *tlc1*Δ-derived type II survivors. EcoRV/SpeI digestion and the TRP1 probe were used for Southern blot. (C) Detection of VII–L telomere changes after recombination-mediated elongation. Three clones (labeled on the top of each panel) for each strain in (A) and (B) were passaged in liquid medium, and Southern blot analysis was performed to examine the telomere length in the cells harvested at the 2^nd^, 8^th^ and 14^th^ passages. EcoRV/SpeI digestion and the TRP1 probe were used. (D) Detection of the length change of total telomeres during liquid passages. The same samples as in (C) were subjected to Southern blot using a TG_1–3_ probe after XhoI digestion to monitor massive telomere signals.

In addition to the distinct bands, the smear signals were detected in the Southern blot membrane (Fig. 1D), suggesting that telomerase-null progenies have chosen different templates as donors to accomplish one round of telomere recombination. In order to further dissect the recombination-mediated telomere extension events, we decided to use the well-established type II survivors to perform HO induction assay. For this experiment, *TLC1* was deleted and then passaged in liquid culture. Cell density was measured and the cultures were diluted to a density of OD_600_ ∼0.05 in fresh medium every 24 hrs. The procedures were repeated 14 times and the well-established type II survivors were obtained. After ∼25 generations of plate growth, a single colony of the type II survivors was inoculated into liquid medium and grown to logarithmic phase, galactose was then added, and the HO endonuclease digestion lasted for 24 hrs. The cells were plated on solid medium until individual colonies arose. Ten independent colonies were randomly picked, their genomic DNA was digested with a pair of restriction enzymes, SpeI and EcoRV, and the telomere length of chromosome VII–L was examined by Southern blot assay using a TRP1 probe (Fig. 1C). In Figure 2A and 2B, both the “uninduced” and “induced” lanes served as controls. Notably, the telomeres in all TG300 clones were shortened compared to the one shown in the induced lane (Fig. 2B). In contrast, the telomeres in all TG81 clones were longer than the induced products (Fig. 2A). Interestingly, the elongated bands in many TG81 clones resembled the size in the uninduced lane (∼2.9 kb), so we wondered if these were HO-uncut or re-ligation products via non-homologous-end-joining (NHEJ) pathway. To solve this doubt, we employed a probe of *MNT2* gene, which is located to the same side of the HO recognition site as *LYS2*, to detect the existence of the *LYS2* arm (Fig. S2A). The Southern blot showed that the “lengthened telomeric fragments” in all the 10 clones of TG81 *tlc1*Δ strain are recombination products (Fig. S2B). Thus, these results indicate that shorter telomeres are more susceptible targets of recombination in type II survivors, and argue against the notion that survivors have acquired a mutation permitting telomere uncapping (and hence telomere recombination) at long telomeres [36].

Previous study has suggested that the recombination event occurring on one single telomere does not trigger the abrupt lengthening of other telomeres once type II survivors are established [21]. We also tested this model in the *de novo* telomere addition system. Three independent clones of each strain shown in Figure 2A–B were passaged in liquid medium, and their VII–L telomere length of passages 2, 8 and 14 was examined by Southern hybridization using the TRP1 probe (Fig. 2C). As expected, both the recombined telomeres (in TG81 clones except No. 9) and the eroded telomeres (in TG300 clones) underwent gradual shortening, and a new round of recombination abruptly took place when a critical telomere length was reached (Fig. 2C). We also detected the total telomere signals between passages 2 and 14 using the TG_1–3_ probe, and found that most of the telomeres were experiencing gradual shortening, which was not keeping pace with VII–L telomere (Fig. 2D). These data strongly supported the notion that in type II survivors, recombination occurring on a shorter telomere does not provoke massive recombination on other telomeres.

**Figure 3:**
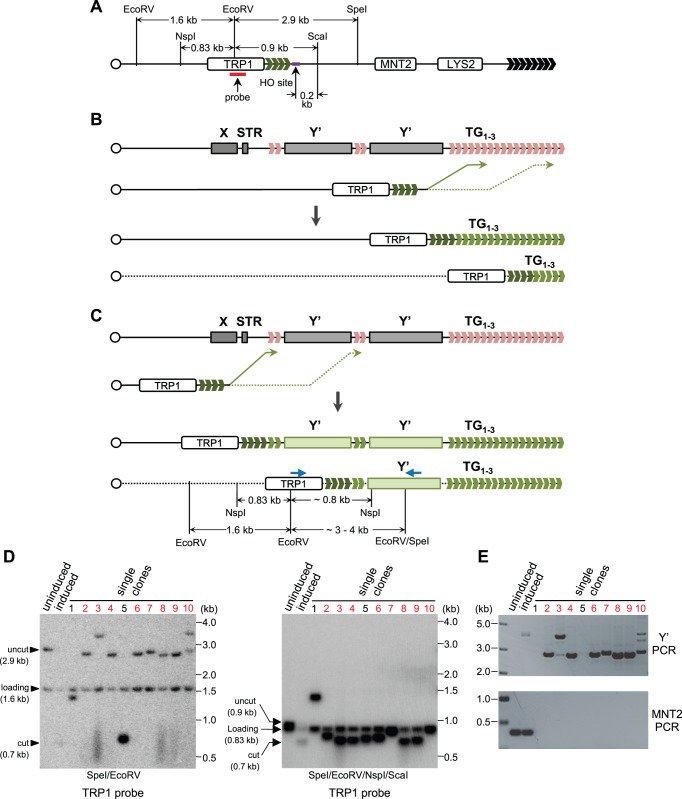
Characterization of VII–L telomere recombination in TG81 *tlc1*Δ type II survivors. (A) Schematic representation of VII–L telomere. The SpeI, EcoRV, NspI and ScaI sites indicated are used for Southern digestion. The HO endonuclease recognition site and the TRP1 probe used for Southern blot are shown in purple and red, respectively. The fragments between the two EcoRV sites (∼1.6 kb) and between the NspI and EcoRV sites (∼0.83 kb) serve as “loading” control respectively for the left and right panels of (D). The fragments digested by EcoRV and SpeI (∼2.9 kb) and by EcoRV and ScaI (∼0.9 kb) indicate the “uncut” positions of Southern signals of the “uninduced” lanes respectively in the left and right panels of (D). The fragments between EcoRV and HO sites (∼0.7 kb) indicate the “cut” positions of Southern signals of the “induced” lanes respectively in the left and right panels of (D). (B) Schematic model of VII–L telomere TG_1–3_ recombination: strand invasion at terminal TG_1–3_ tracts of other telomeres generates products of various lengths with TG_1–3_ sequence. (C) Schematic model of VII–L telomere recombination involving copying Y′-element: strand invasion at subtelomeric TG_1–3_ tracts of other telomeres generates ∼3–4 kb products upon SpeI/EcoRV digestion or ∼0.8 kb products upon SpeI/EcoRV/NspI/ScaI digestion. Positions of the primers used for Y′-PCR are indicated with blue arrows. The figures in (A), (B) and (C) are not precisely drawn to scale. (D) Southern blot identification of the recombination products containing Y′ sequence. SpeI/EcoRV (left panel) or SpeI/EcoRV/NspI/ScaI (right panel) were used for Southern digestion. The TRP1 probe was used for Southern hybridization. The 1.6 kb (left panel) and 0.83 kb (right panel) fragments serve as the loading control. (E) Upper panel: PCR analysis of the recombination products containing Y′ sequence. Sequences of primers for Y′-PCR are: 5′-AATGCCGTAATCATTGACCA-3′ (from *TRP1* ORF) and 5′-TAAGCGCAACTGATGCAAGT-3′ (from Y′ element). Lower panel: PCR analysis of the successful HO-induction. Sequences of primers for *MNT2* PCR are 5′-CTATGGGTGTATTGGGCTTG-3′ and 5′-GACCCGTCCCTGTCTGTATG-3′.

We noticed that in TG81 *tlc1*Δ strain, 8 out of 10 clones generated the recombination products which were uniformly around 3 kb when the genomic DNA was digested with SpeI and EcoRV (Fig. 2A). The 3 kb band in clone 9 of TG81 *tlc1*Δ strain appeared not to be shortened during sequential passages (Fig. 2C), suggesting that these ∼3 kb restriction fragments might not be the results of stochastic recombinations between the short VII–L telomere and the terminal TG_1–3_ tracts in other telomeres (see the schematic model in Fig. 3B). Because there are short TG_1–3_ tracts between subtelomeric X and Y′ elements, as well as two Y′ elements (Fig. 3B), it is possible that the ∼3 kb bands seen in TG81 *tlc1*Δ strain are resulted from copying Y′ element (see the schematic model in Fig. 3C). To test this model, we randomly picked 10 clones of the TG81 *tlc1*Δ cells, extracted their genomic DNA, and used two groups of restriction enzymes respectively, SpeI/EcoRV and SpeI/EcoRV/NspI/ScaI, to digest the DNA. The combination of SpeI/EcoRV/NspI/ScaI restriction enzymes can cut Y′ sequence at multiple sites, generating fragments with the size of shorter than 1 kb, while leave the TRP1-TG_1–3_ fragment on the induced end intact. As a result, the recombination products resulting from terminal TG_1–3_ tracts may appear at various lengths and give rise to identical bands upon SpeI/EcoRV and SpeI/EcoRV/NspI/ScaI treatments (Fig. 3B), whereas the Y′-containing recombination products display ∼3–4 kb bands after SpeI/EcoRV digestion but migrate to ∼0.8 kb after SpeI/EcoRV/NspI/ScaI digestion (Fig. 3C). Then we used the TRP1 probe to perform Southern blot assays and found that SpeI/EcoRV digested DNA fragments with the size of ∼3–4 kb (Fig. 3D, lanes marked in red in the left panel) disappeared, and the fragments with the size of 0.7–0.8 kb showed up upon additional NspI and ScaI digestion (Fig. 3D, right panel), suggesting that the clones exhibiting ∼3–4 kb signals have copied Y′ sequence onto the induced short telomere ends (Fig. 3C). The recombinational bands shown with SpeI/EcoRV digestion in clone 1 and 5 were not sensitive to the digestion of NspI and ScaI (Fig. 3D, compare left and right panels), and they were likely produced by copying different lengths of TG_1–3_ tracts (Fig. 3B). To further validate that the ∼3–4 kb signals on the SpeI/EcoRV membrane contain Y′ sequence(s), we carried out a PCR experiment using both Y′ and TRP1 specific primers (the positions of which are labeled by blue arrows in Fig. 3C), and detected very specific signals in the clones which had ∼3–4 kb bands in the SpeI/EcoRV treated DNA (Fig. 3E, upper panel). Consistently, the PCR analysis using *MNT2* primers illustrated that all the ∼3–4 kb bands were not HO-uncut or NHEJ products (Fig. 3E, lower panel). Taken together, our results suggest that the short VII–L telomeres can initiate recombination by invading both TG_1–3_ tracts being internal to Y′ sequence (Fig. 3C) and the very terminal TG_1–3_ sequence of other telomeres (Fig. 3B).

### Efficient Telomere Recombination Requires Rad50, Rad59 and Cgi121 in Type II Survivors

**Figure 4:**
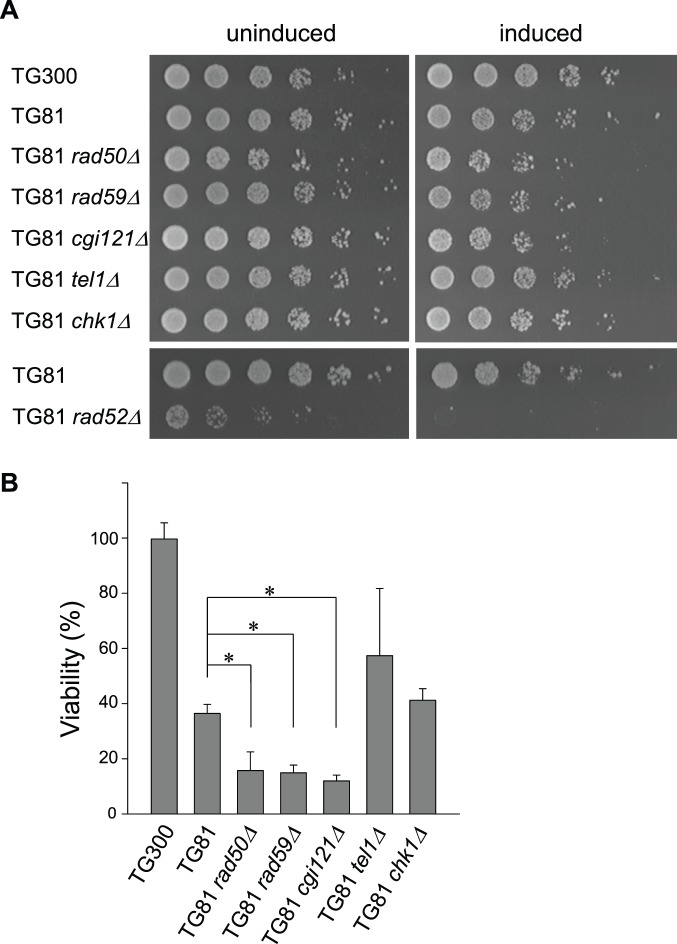
Viability assays. All the mutants were derived from the TG81 *tlc1*Δ type II survivors (TG81 for short), and TG300 *tlc1*Δ type II survivors (TG300 for short) served as a control. (A) Dotting assay. Cell cultures were diluted with sterile water to a concentration of OD_600_ ∼0.3, and 3 µl of each 5-fold serial dilutions was dotted on YC plates. Plates were incubated at 30°C for 2 to 3 days and then photographed. *rad50*Δ and *cgi121*Δ are mutants of slow growth, which were dotted earlier than other strains. (B) Quantification of viability assays. 100 µl of the 5^th^ dilution sample in (A) was plated. Plates were incubated at 30°C for 2 to 3 days and colony numbers were counted. The viability was calculated using the formula: Viability = (Induced total - Induced uncut)/Uninduced total. Error bars represent standard deviations of three independent experiments. Compared to TG81 control, P values of the *rad50*Δ, *rad59Δ*, *cgi121*Δ, *tel1Δ* and *chk1Δ* mutants are 0.030, 0.025, 0.015, 0.295 and 0.317, respectively. Significance (*) is defined by P value <0.05.

It is now widely accepted that in *S. cerevisiae*, Rad50 and Rad59 are essential for the type II survivor formation, Rad51 is essential for the type I survivor formation, while Rad52 is required for the generation of both types of survivors in telomerase deficient cells [13], [14], [17]. Cgi121 is a subunit of yeast KEOPS complex, which is indispensable for type II survivor generation [22]. Therefore, we suspected that recombination could not occur in the type II survivors lacking *RAD52*, *RAD50*, *RAD59* or *CGI121*. We knocked out *RAD52*, *RAD50*, *RAD59* or *CGI121* in the *tlc1*Δ-derived type II survivors, and performed HO induction assay followed by dotting assay to examine the cell viability in these mutants. If there was no 81-bp short telomere induced by HO endonuclease, all the strains showed regular growth except that the *tlc1*Δ *rad52*Δ mutant exhibited much lower viability than others (Fig. 4A, uninduced panel). When an 81-bp short telomere was created, the *tlc1*Δ *rad52*Δ mutant died immediately, and the *tlc1*Δ *rad50*Δ, *tlc1*Δ *rad59*Δ and *tlc1*Δ *cgi121*Δ mutants displayed considerably reduced viability (Fig. 4A, induced panel). These results indicated that Rad52 is absolutely required for repairing shortened telomeres [16], while Rad50, Rad59 and Cgi121 are not essential for, but facilitate telomere recombination.

Since critical short telomere(s) triggers cell cycle arrest and cell death if such short telomere(s) is not repaired by recombination, we used a plating assay to examine the cell viability, which is able to quantify the recombination efficiency on the short telomere of VII–L induced by HO endonuclease. In the TG300 strain, nearly 100% of the cells were viable upon HO induction, providing a control for other strains (Figs. 4A and 4B). The viability of the TG81 strain was 36% (Fig. 4B), because only the cells that recombine their short telomeres could be selected to survive. The recombination efficiency of the *rad50*Δ, *rad59*Δ and *cgi121*Δ mutants were 16%, 15% and 12%, respectively, which were significantly lower than that of the TG81 control (Fig. 4B).

Previous studies demonstrated that in telomerase positive cells, Tel1 preferentially associates with short telomeres to stimulate their elongation by telomerase [26]–[28], and Tel1 participates in the generation of type II survivor [23]. In the well-established type II survivor cells, deletion of *TEL1* did not reduce the recombination efficiency (Figs. 4A and 4B), suggesting that Tel1 functions in the steps of generating type II survivors, but not in the period of type II survivor maintenance. It will be of interest to perform an extensive study on whether the genes required for type II survivor generation are still indispensable in type II survivor maintenance [22]. We also explored the telomere recombination efficiency in the mutant of another DNA damage checkpoint protein, Chk1, and obtained similar results as seen in *tel1*Δ mutants (Figs. 4A and 4B).

### Rad50, Rad59 and Cgi121 are not Essential for Telomere Recombination in Type II Survivors

**Figure 5:**
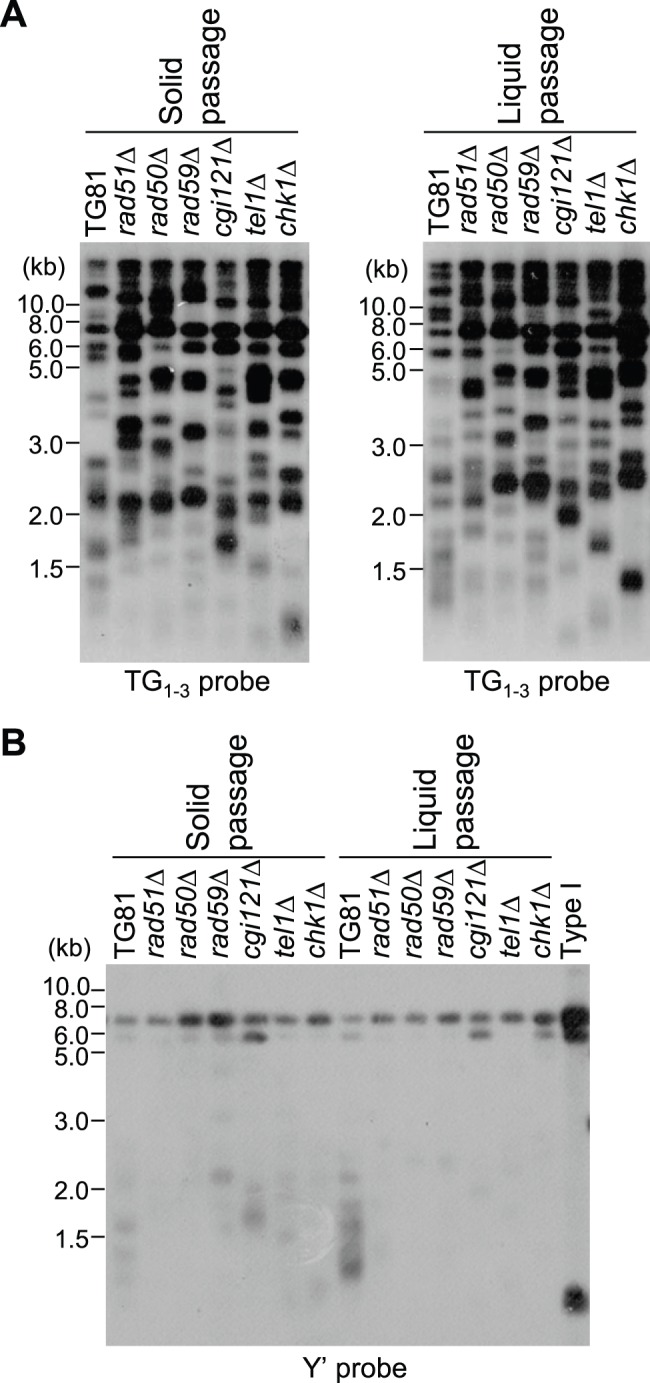
Examination of survivor type. (A) Cells were passaged on solid medium by successive restreak for ∼300 generations (left panel), and in liquid medium by diluting to a cell density of OD_600_ ∼0.05 every 24 hrs for 14 times (right panel). Southern blot was performed using a TG_1–3_ probe after XhoI digestion of genomic DNA. All the mutants were derived from the TG81 *tlc1*Δ type II survivors (TG81 for short), and *rad51*Δ served as a control. (B) Southern blot with the same samples as in (A) was performed using a Y′ probe after XhoI digestion of genomic DNA. Typical type I survivors served as a control.

Since Rad50, Rad59 and Cgi121 are required for type II survivor generation, it remains possible that upon the inactivation of either Rad50, Rad59 or Cgi121, the *tlc1*Δ type II survivors might be forced to activate type I recombination, and switched to type I survivors. To test this possibility, we passaged the *tlc1*Δ *rad50*Δ, *tlc1*Δ *rad59*Δ and *tlc1*Δ *cgi121*Δ type II survivors on both solid and liquid media for 250 to 300 generations, and examined their telomere structures by Southern blot analysis. The results showed that the telomeres in all these mutants were of typical type II, and no significant Y′ amplification was detected (Figs. 5A and 5B).

**Figure 6:**
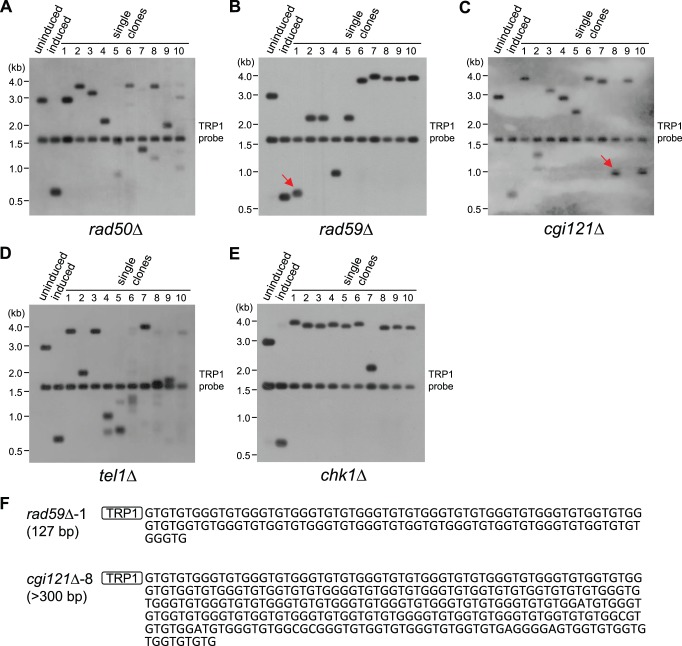
Examination of VII–L telomere recombination in the *rad50*Δ, *rad59*Δ, *cgi121*Δ, *tel1*Δ and *chk1*Δ mutants. All the mutants were derived from the TG81 *tlc1*Δ type II survivors. HO induction assay and Southern blot analysis (EcoRV/SpeI digestion, TRP1 probe) were performed in (A) *rad50*Δ, (B) *rad59*Δ, (C) *cgi121*Δ, (D) *tel1*Δ and (E) *chk1*Δ mutants. (F) Representive sequencing results of VII–L telomere in *rad59*Δ-1 and *cgi121*Δ-8 (Figs. 6B and 6C, indicated with arrows) type II survivor cells.

To further verify the existence of recombination-mediated elongation of short telomeres in the *tlc1*Δ *rad50*Δ, *tlc1*Δ *rad59*Δ and *tlc1*Δ *cgi121*Δ type II survivors, we performed HO induction assay and examined the VII–L telomeres in ten independent clones. The Southern blot results with both a *TRP1* and a *MNT2* hybridization probe revealed that recombination took place on the HO-induced VII–L telomeres in the *rad50*Δ, *rad59*Δ and *cgi121*Δ mutants, except that *rad50*Δ-1 clone was not successfully cut by HO (Figs. 6A–C and S3). We then performed VII–L telomere PCR in the samples of *rad59*Δ-1 and *cgi121*Δ-8 (Figs. 6B and 6C, indicated with arrows), and the sequencing data confirmed that the telomere recombination products resulted from TG_1–3_ amplification (Fig. 6F). Thus, we concluded that Rad50, Rad59 and Cgi121 are not essential for telomere recombination in type II survivors [17]. In addition, the telomere recombination events were also observed in the *tel1*Δ and *chk1*Δ mutants (Figs. 6D, 6E and S3).

**Figure 7:**
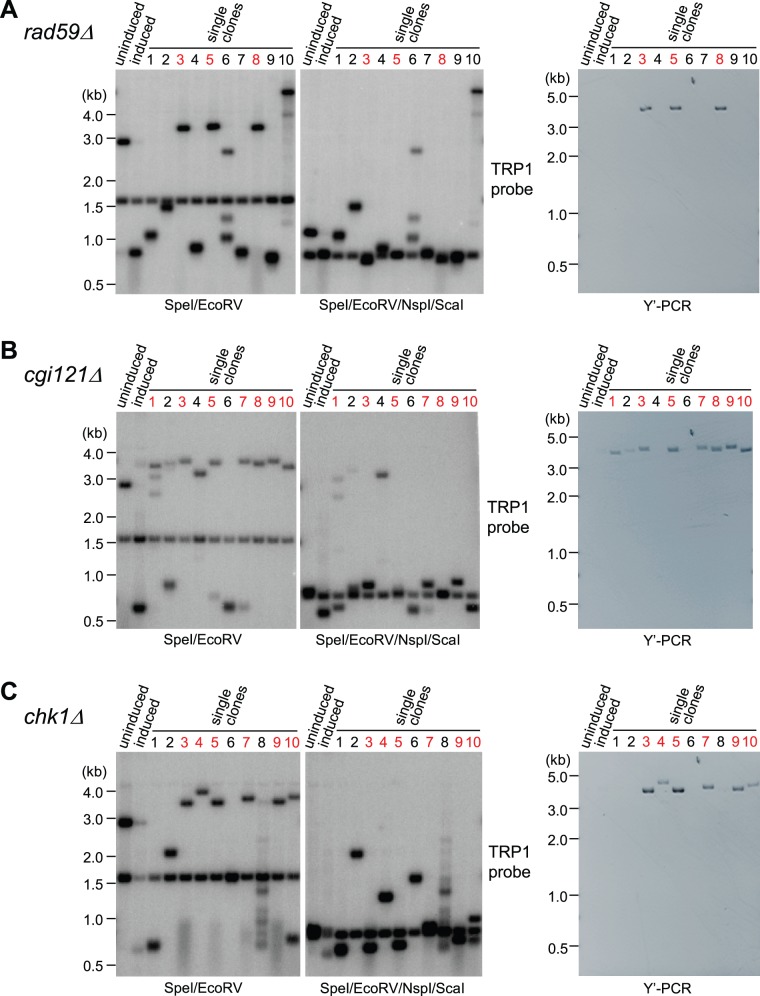
Characterization of VII–L telomere recombination in the *rad59*Δ, *cgi121*Δ and *chk1*Δ mutants. All the mutants were derived from the TG81 *tlc1*Δ type II survivors. HO induction assay, Southern blot (left panels) and PCR analyses (right panels) were performed in (A) *rad59*Δ, (B) *cgi121*Δ and (C) *chk1*Δ mutants. SpeI/EcoRV or SpeI/EcoRV/NspI/ScaI digestion and the TRP1 probe were used for Southern blot. The 1.6 kb (SpeI/EcoRV panels) and 0.83 kb (SpeI/EcoRV/NspI/ScaI panels) fragments serve as the loading control. Y′-PCR was performed as described in Figure 3E.

In addition to the fragments that are shorter than 3 kb, some larger fragments of ∼3–4 kb were also detected in the *rad50*Δ, *rad59*Δ, *cgi121*Δ, *tel1*Δ and *chk1*Δ mutants of TG81 *tlc1Δ* type II survivors (Figs. 6A–E). To evaluate whether those recombination events involved copying Y′ element (Fig. 3C), we randomly chose ten more clones of TG81 *tlc1*Δ *rad59*Δ, TG81 *tlc1*Δ *cgi121*Δ, TG81 *tlc1*Δ *chk1*Δ mutants, in which HO-induced short VII–L telomere had experienced recombination-mediated elongation. The Southern blot experiments showed that most of the ∼3–4 kb bands were sensitive to the digestion of NspI and ScaI, while all the shorter bands (<3kb) were not sensitive to the digestion of NspI and ScaI (Fig. 7, panels labeled SpeI/EcoRV/NspI/ScaI). The PCR analyses revealed that most of the long telomere VII–L had copied Y′ sequence (Fig. 7, lanes marked in red in the panels labeled Y′-PCR). Notably, the clone 10 of the TG81 *tlc1*Δ *rad59*Δ mutant (Fig. 7A) and the clone 4 of the TG81 *tlc1*Δ *cgi121*Δ mutant (Fig. 7B) had long teminal TG_1–3_ sequence because their telomere VII–L were not sensitive to SpeI/EcoRV/NspI/ScaI digestion. These results support both models shown in Figure 3B and 3C: a critical short telomere in a type II survivor can invade into the TG_1–3_ homologous sequence either at subtelomeric region to copy Y′-containing sequence, or at the terminal telomeric region to copy TG_1–3_ sequence.

## Discussion

It is well known that short telomeres are preferred substrates for telomerase [25]. We wonder whether this preference also exists in the process of recombination-mediated telomere elongation. In this study, we have shown that in *Saccharomyces cerevisiae*, short telomeres are preferentially elongated by recombination in the senescing telomerase-null pre-survivors (Figs. 1A and 1B). Our result is consistent with that shown in a previous study by Chang et al, who also found that in *S. cerevisiae*, short telomeres in pre-survivor cells are more likely to be extended by recombination [29]. Furthermore, McEachern and Topcu studied the recombinational telomere elongation events in another kind of yeast, *Kluyveromyces lactis*, and demonstrated that recombination is greatly induced near shortened telomeres [37], [38]. Interestingly, Chang et al also followed the growth of the *est2Δ* cultures to identify the senescence point, and showed a recombination preference for long telomeres during the emerging stage of survivors [29]. However, their approach could not tell whether long or short telomeres are the preferred targets of recombination during the maintenance stage of type II survivors, because telomeres of several kb in length, which are common in type II survivors, can hardly be recovered in telomere sequencing assay. We employed the HO induction system and assayed telomere length (both gross telomere signals and single telomere of VII–L) by Southern blot analysis. In the established type II survivors, the short VII–L telomeres in the TG81 clones have recombined to generate longer ones, whereas the 300-bp long VII–L telomeres in TG300 clones have experienced shortening before any recombination event occurs (Figs. 2A and 2B). Previously Teng et al reported that in the established type II survivors, the long VII–L telomeres did not undergo recombination-mediated lengthening, but rather shortened during ∼350 cell divisions, and an abrupt elongation took place once the telomeres shortened to a critical length [21]. Taken together, these observations indicate that short telomeres are the preferential substrates for recombination in both pre-survivors and established type II survivors. Due to the survival pressure cells preferentially lengthen short telomeres because senescence would be induced if the critically short telomeres are not repaired. Thus, even in the emerging survivors that prefer long telomeres, the very short telomeres were also elongated by recombination [29]. In pre-survivors, the newly added sequences seem to be very short (Figs. 1A and 1B), while in type II survivors, telomeres elongated by recombination could reach several kb (Fig. 2). These results suggest that in order to maintain certain telomere length, pre-survivors and type II survivors might use different recombination mechanisms, i.e. copying an eroded short telomere and replicating from a template like t-circle to generate a long telomeric sequence respectively, although they all exhibit the preference for short telomeres. However, we cannot exclude the possibility that the long recombination products in pre-survivors (Figs. 1A and 1B) were not retrievable in telomere sequencing assay due to technical difficulties. To this end, we propose the model that recombination machinery favors different telomere substrates at different stages: in pre-survivors and established type II survivors, recombination occurs on short telomeres (this study), while long telomeres are preferentially elongated during the type II survivor formation [29]. We do not know why shorter telomeres are more susceptible to homologous recombination at the stages of pre-survivors and established type II survivors, while longer ones are more favorable at the emerging stage of type II survivors. One possibility is that in pre-survivors and established type II survivors, the shorter telomeres are less protected than the longer ones; but in in emerging survivors, the longer telomeres are less protected than the shorter ones. Less protected telomeres might facilitate single-strand invasion, a critical step of homologous recombination. The other possibility is that t-circles are only generated in the emerging stage, and they are more accessible by longer telomeres. A third possibility is that during the critical phase of survivor formation, gross telomere recombination is activated, and longer telomeres are preferred because they provide more and better substrates for recombination. These speculations need experimental evidence to support.

In the established type II survivors, recombination occurring on a shorter telomere does not provoke massive recombination on other telomeres (Figs. 2C and 2D) [21]. In contrast, the HO-cut short VII–L telomere in the TG81 *tlc1Δ* cells seems to be able to trigger more rapid gross telomere recombination because the formation of type II survivors in the TG81 *tlc1Δ* cells likely emerged earlier than that in the TG300 *tlc1Δ* cells (Figs. 1D and S1). These results suggest that longer telomeres might be involved in recombination during survivor formation, and support the notion raised by Chang et al that recombination prefers long telomeres during the emerging stage of survivors [29].

It is generally accepted that both Rad50 and Rad59 are required for type II survivor generation [17]. In the established type II survivors, inactivation of either Rad50 or Rad59 did not prevent recombination from taking place on the HO-induced VII–L telomeres (Figs. 6A and 6B). It is surprising because our results seem to be contradictory to the well-documented role of Rad50 and Rad59 in type II survivor generation [17]. We thought that the discrepant conclusions might be attributed to the differences between the two assays. In the telomerase-null senescing cells, the 32 telomeres all undergo shortening until to the critical length, resulting in cell cycle arrest [39], [40]. In order to generate type II survivor(s), presumably multiple (if not all of the 32) critical short telomeres must experience the repair process via recombination. That’s why even under the conditions that all the factors involved in recombination are present, the probability of generating a type II survivor is still very low (<10^6^) [20]. Therefore, the high recombination efficiency, granted by Rad52 group factors (including Rad50 and Rad59), is crucial for the survivor generation, and it is not surprising to see that both Rad50 and Rad59 are required for type II survivor formation. However, in an established type II survivor, most of the telomeres are long. To maintain the viability of a type II survivor, only the critical short telomere(s), which account for a few, need to be elongated. Inactivation of Rad50 or Rad59 may cause a problem, but it might not result in catastrophic defect to the viability of a type II survivor.

## Supporting Information

Figure S1 **Examination of massive telomere recombination in senescing** ***tlc1Δ*** **cells.** The same samples of genomic DNA as in Figure 1D were digested by XhoI and Southern blot was performed using a TG_1–3_ probe.
(EPS)

Figure S2 **Examination of existence of the** ***LYS2*** **arm.** (A) Schematic representation of the *MNT2* marker used for Southern blot detection. EcoRI and SalI indicated were used for Southern digestion and a 387 bp fragment (shown in gray) within *MNT2* was used as the probe. This figure is not precisely drawn to scale. (B) Southern blot examination of *LYS2* arm loss upon HO-induction in TG81/300 *tlc1Δ* type II survivors.
(EPS)

Figure S3 **Examination of existence of the** ***LYS2*** **arm in** ***rad50*** **Δ,** ***rad59*** **Δ,** ***cgi121*** **Δ,** ***tel1*** **Δ and** ***chk1*** **Δ mutants of TG81** ***tlc1Δ*** **type II survivors.** EcoRI and SalI were used for Southern digestion and the MNT2 probe was used for hybridization.
(EPS)

Sequence S1 **Telomere I–L sequencing results of** ***est2*** **Δ strain.**
(TXT)

Sequence S2 **Telomere I–L sequencing results of** ***est3*** **Δ strain.**
(TXT)
